# Quantification of Defensive Proteins in Skin Mucus of *Atlantic salmon* Using Minimally Invasive Sampling and High-Sensitivity ELISA

**DOI:** 10.3390/ani10081374

**Published:** 2020-08-07

**Authors:** Haitham Tartor, Adérito Luis Monjane, Søren Grove

**Affiliations:** 1 Fish Health Research Group, Norwegian Veterinary Institute, P.O. Box 750, Sentrum, N-0106 Oslo, Norway; haitham.tartor@vetinst.no (H.T.); aderito-luis.monjane@vetinst.no (A.L.M.); 2 Institute of Marine Research, P.O. Box 1870, 5817 Bergen, Norway

**Keywords:** *Atlantic salmon*, skin mucus, gill mucus, minimally invasive sampling, specific IgM antibodies, complement component 5, sensitive ELISA

## Abstract

**Simple Summary:**

The external surfaces of fish, including the skin, are covered by mucus. Mucus is an important and multifunctional matrix of substantial complexity. The mucus is viscous and sticky, and adheres to the underlying epithelium, making the sampling of mucus challenging. To help define more standardized protocols for mucus sampling, we here compare three different sampling methods. The methods include scraping of mucus, wiping of mucus, and absorption of the liquid part of mucus. We compare the resulting damage to the fish skin and compare the content of two specific immune proteins in the three sample types. Using histological examination, we show that absorption leads to very limited damage to the skin epithelium while scraping causes substantial damage. Using Enzyme-Linked Immunosorbent Assay (ELISA) methods, we show that the mucus sample types contain similar amounts of antigen specific immunoglobulin M (IgM) and complement component 5 (C5), respectively. The levels of the IgM but not the C5 were moderately correlated between mucus and blood from the same fish, suggesting the importance of fish skin mucus for analyzing antigen-specific IgM after vaccination procedures. We conclude that absorption is an easily performed and minimally invasive sampling method that produces mucus samples with comparable contents of IgM and C5.

**Abstract:**

Protocols used to collect fish skin mucus may inadvertently compromise the sampled fish or the resulting sample. Here, we used three methods (wiping, scraping, and absorption) to collect skin mucus from Atlantic salmon and compared their invasiveness on fish skin epithelium. We found that the absorption method was the least invasive. We also compared the abundance of antigen-specific immunoglobulin M subtype A antibodies (IgM-A Ab) and complement component 5 (C5) in mucus samples collected from vaccinated fish by the three methods. An enzyme-cascade-amplification strategy colorimetric immune assay was optimized and used to analyze IgM-A, and ELISA was used to analyze C5. The abundance of antigen-specific IgM-A in skin mucus was comparable between the three methods, but C5 was significantly lower in absorbed mucus in comparison to in the wiped or scraped mucus samples. Absorbed skin mucus samples collected from various body regions of salmon, levels of C5 were comparable, while specific IgM-A amounts varied between the regions. By comparing three mucus-absorbing materials (medical wipe, gauze, and cotton) for their ability to absorb and release IgM-A and C5, medical wipes proved to be ideal for IgM-A analysis, whereas gauze was the best for C5 analysis.

## 1. Introduction

Mucus is a gelatinous matrix covering membranes associated with epithelial surfaces and can be organized into two distinct layers: an inner viscous layer that spans a thin region which is firmly attached to the epithelial cells, and above, with an unattached and thicker layer with high water content [1]. In fish, skin mucus is crucial for the regulation of physiological processes such as respiration, excretion, ionic and osmotic regulation, and as the first line of defense against various pathogens [2], partly because of the different immune factors within it [3]. Given its multifunctional nature, it is unsurprising therefore that there is a great deal of interest in sampling and studying mucus, especially in economically valuable fish. However, one major caveat in fish mucus research has been the very methods used to collect it [4]. Various studies have used so-called “scraping” [5], “swabbing/wiping” [6] or “massaging of fish in a plastic bag” [7] methods to collect skin mucus, and each method has been associated with its set of challenges. These challenges may include difficulties in limiting the sampling to specific sites or controlling the extent of sample dilution that occurs when using the method. Crucially, however, the different methods may cause a certain degree of damage to the epithelium (dermal layer) from which the mucus is sampled. In cases where repeated sampling of mucus from the same fish is required, it would clearly be beneficial to use a mucus sampling method that causes minimum alteration to the dermal layer during sampling. The use of such a method would be consistent with the three ethical principles (replacement, reduction, and refinement [8]) of using fish as a laboratory animal in research.

In an attempt to investigate what constitutes an optimal mucus sampling method, two variants of a method that absorbs the liquid part of fish skin mucus were described [9,10]. Both approaches attempt to leave most of the viscous matrix on the fish skin surface undisturbed by sampling mucus as follows: pieces of absorbent material are placed on the skin of the fish for a few seconds until saturated with mucus liquid, and then gently removed for further processing. The comparatively less invasive nature of this “absorption” method had an additional advantage. A metabolomics [10] and proteomics [11] analysis of mucus sampled by the absorption method proved it to be both qualitatively and quantitatively comparable to mucus sampled by either the scraping or wiping method, with the additional benefit that the absorbed mucus samples showed the least inter-individual variation in metabolites [10], and contained the least abundance of cellular-derived proteins when compared to the other two methods [11]. The latter observation suggests again that in the process of sampling the liquid part of skin mucus, the absorption method least disturbs the epithelial layer supporting the mucus matrix and consequently fewer cellular proteins end up in the resulting mucus sample.

The distinction between cellular proteins from the epithelial layer, and proteins present in the mucus matrix is important, especially when assessing the immunological properties of fish skin mucus. Among the topical immunological molecules are complement components (CCs) and secretory immunoglobulins (sIgs). Complement components are innate immune molecules that play a pivotal role in the inflammation process as part of the innate defense against different pathogens and contribute significantly to the adaptive immune response against them [12]. Among these, complement component 5 (C5) is central in chemotaxis events occurring during inflammation, and to the assembly of the membrane attack complex [13]. Secretory immunoglobulins are the key effectors of the adaptive humoral immune response [14]. In fish, sIgs include sIgD [15], sIgT [16] and sIgM, with the latter being the dominant systemic antibody.

In fish, CCs and sIgs are typically analyzed using blood samples, where acquisition of the sample often requires termination of the fish. However, since CCs and sIgs are present in fish skin mucus [17,18]—although sIgs are in relatively low concentrations [16,19]—the use of sufficiently sensitive methods should allow for analyses of these molecules in fish in a less invasive manner. An “enzyme-cascade-amplification strategy colorimetric immune assay” (ECAS-CIA) was recently developed to measure concentrations of low-abundance proteins in human serum [20]. The increased sensitivity of the ECAS-CIA comes from adding an amplification step that employs the powerful catalytic activity of palladium nanostructures (PdNS) [21] to a conventional Enzyme-Linked Immunosorbent Assay (ELISA) protocol. Adaptation of the ECAS-CIA to analyses of fish skin mucus may render it possible to analyze low-abundance sIgs.

The present study follows two research publications that compare mucus-sampling protocols, with the ultimate aim of defining the least invasive method capable of delivering mucus samples that best represent “true” mucus—that is, mucus unaffected by dermal damage. In this trilogy of papers, mucus sampled by scraping, wiping, and absorption methods were previously compared using metabolomics [10] and proteomics [11] approaches. Here, quantitative methods for two specific immune proteins were used to compare the exact same mucus samples used in studies [10,11]. Histological analysis was applied to compare tissue damage in samples of skin that had been subjected to the three methods. Furthermore, we compared some aspects of the mucus absorption method including: (i) the choice of material used for mucus absorption; (ii) mucus absorption from different skin areas of fish; (iii) and the viability of this method to collect mucus from salmon gills. A sensitive ECAS-CIA assay was also adapted and used to analyze immunoglobulin M subtype A (IgM-A) in the absorbed mucus. Altogether, our results showed that of the three mucus-sampling methods tested, the absorption method had the least impact on fish skin epidermis, and it could be used to sample gill mucus. We found also that IgM-A and C5 could be detected in skin mucus irrespective of the sampling method used. Interestingly, the IgM-A and C5 were observed to vary in their abundance in absorbed samples according to the skin area sampled, or the absorption material employed. Overall, we believe that the minimally invasive nature of the absorption method makes the method a considerably more 3R-friendly approach (by reducing the number of animals required) that could be adopted to sample fish skin mucus for various assays.

## 2. Materials and Methods

### 2.1. Ethics Statement

This study was conducted at the Norwegian Institute for Water Research (NIVA) in Drøbak. The Norwegian Animal Research Authority approved these experiments (FOTS IDs: 12009), and all of our procedures on fish were implemented according to the Norwegian Acts and Regulations regarding the use of fish in experimentation.

### 2.2. Fish and Vaccination

**Figure 1:**
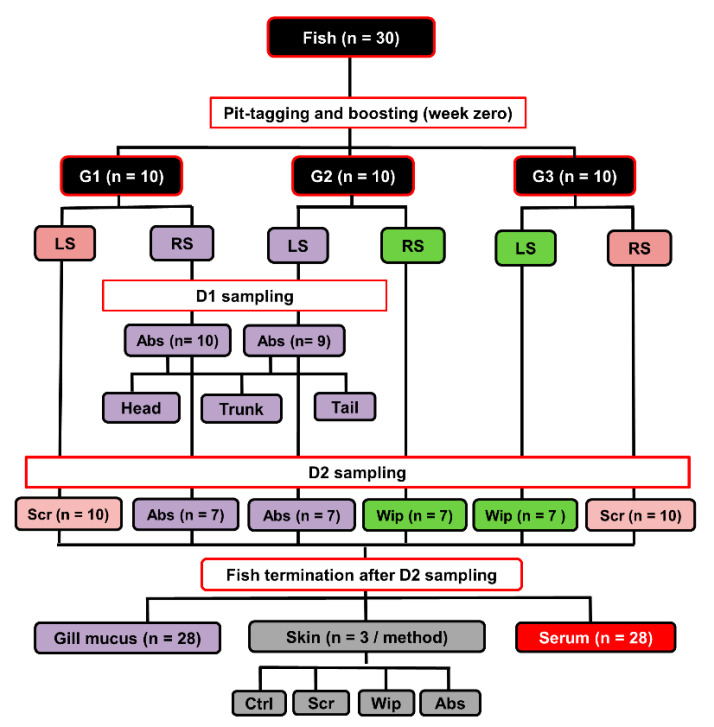
Schematic representation of the experiments conducted at the research aquaria facility, the figure shows how the total number of fish/samples were sub-divided and used in smaller experiments. Mucus samples collected by the same method have the same box color on the diagram. Fish were vaccinated at the beginning of the experiment and were sampled at two time-points (12- and 18-weeks post-vaccination). G: group, LS: left side, RS: right side, Ctrl: control, Scr: scraping, Abs: absorption, Wip: wiping, wpv: weeks post-vaccination. D1: First skin mucus sampling (12 wpv), D2: Second skin mucus sampling (18 wpv).

Atlantic salmon fish (*Salmo salar*; *n* = 30) weighing between 1–2 kg was held in fiberglass tanks (1 m^3^) supplied with running seawater with a salinity average of about 33‰ and at a water temperature of 10 °C on average. Big fish were chosen to ensure that sufficient volumes of skin mucus could be sampled from both right and left sides. Fish were intraperitoneally vaccinated with ALPHA JECT micro 6^®^ vaccine (100 µL/fish; PHARMAQ AS, Oslo, Norway), which contains antigens from five bacteria (*Aeromonas salmonicida*, *Listonella anguillarum* (serotype O1 and O2), *Vibrio salmonicida*, *Moritella viscosa*) and one virus (*Infectious pancreatic necrosis virus*). All fish were pit-tagged to allow repeated sampling from the same individual. A complete overview of the experimental design, number of fish used, and numbers of collected samples is shown in Figure 1.

### 2.3. Skin Mucus Sampling Procedures

Fish were sampled twice for their skin mucus at 12- and 18-weeks post-vaccination. These time points/samples will be referred to as “D1” and “D2”, respectively. Only fish with apparently healthy skin and clear mucus were used, whereas fish with any external lacerations or with blood-contaminated mucus were excluded. Before sampling, fish were sedated with 10 mg/mL benzocaine (BENZOAK, ACD Pharmaceuticals AS, Oslo, Norway) in their holding tanks to facilitate their capture and to limit excessive wriggling that otherwise could have disturbed the integrity of the mucus layer. Fish were subsequently anesthetized with 200 mg/mL benzocaine in a separate tank, identified by their pit-tags and held by the mouth and caudal fin base to allow water to run off. Fish were positioned on their ventral side, and mucus was subsequently obtained from both the right and left lateral sides as described below.

Three different mucus sampling methods (absorption, wiping, and scraping) were used in a pairwise fashion on individual fish to collect skin mucus sample pairs (D2; *n* = 30). The fish were randomly divided into three groups (Groups 1−3), with ten fish per group. From each fish in Group 1, mucus was sampled by scraping from one lateral side and by wiping from the opposite lateral side. In Group 2, the sampling methods used were scraping and absorption (again, on opposite sides), and in Group 3 absorption and wiping. Mucus samples accidentally contaminated with blood or feces were excluded from the study. This resulted in twenty scraped, fourteen wiped, and fourteen absorbed mucus samples (Figure 1). Below is a brief description of each mucus-sampling method. The absorption method was performed as described by Ivanova et al. [10]. Briefly, fish (*n* = 20 from D1 and D2) were placed on their ventral side, and skin mucus was absorbed by covering most of the surface, i.e., from caudally of the eye to cranially of the tail fin with pieces of medical wipes (2.5 × 7 cm each; Kimberly-Clark, Kent, UK). The wipes were left on fish for approximately 15 s until they were completely wet with mucus. After absorption, all individual pieces were gently removed using forceps, and each was then placed into the upper compartment of a 0.45 µm pore size Corning^®^ Costar^®^ Spin-X^®^ polypropylene centrifuge tube (in short, “Spin-X tubes”; Sigma-Aldrich, Salt Lake City, UT, USA). The Spin-X tubes were then kept on ice for less than 2 h before being centrifuged at 13,000× *g* (4 °C, 10 min) to produce a watery, non-viscous mucus filtrate. These filtrated mucus samples were then stored at −80 °C until further analysis. For wiping, the lateral side assigned to this method (D2; *n* = 20) was wiped from head to tail, replacing the medical wipes with fresh ones as the previous ones became saturated. After that, the mucus-saturated wipes were separately placed into Spin-X tubes and treated as described above. The scraped mucus samples were collected (D2; *n* = 20) as previously described [10] by scraping off the mucus with the foil the scalpel blades come wrapped in a head to tail direction. The scraped samples were placed into Spin-X tubes and treated as already described. The skin mucus samples collected by the absorption method had on average lower volumes (1.5 mL/fish) as compared to that collected by scraping and wiping methods (3 and 5 mL/fish, respectively).

### 2.4. Sampling of Gill Mucus, Blood and Skin

Gill mucus, blood, and skin samples were also collected at D2. A modified version of the absorption method was used to collect mucus from fish gills. Briefly, a single piece of medical wipe (2 × 2 cm) was placed for a few seconds (long enough for the filters to become wet, but not so long that gill epithelium was inadvertently collected) on the lateral surface of every gill filaments in both the left and right gill of each fish (D2; *n* = 28). The mucus-containing wipes were then processed as described earlier.

Before collecting blood and skin samples, fish were euthanized by an overdose of BENZOAK. Blood samples were collected from the caudal vein of euthanized fish (D2; *n* = 28) using vacutainer tubes (VACUETTE^®^, Greiner Bio-One, Frickenhausen, Germany). Samples were allowed to clot overnight at 4 °C and then centrifuged (2000× *g*, 4 °C, 5 min) to isolate the serum. These were then stored at −80 °C until analyzed.

Skin samples were collected from the occipital head region of euthanized fish immediately after mucus had been sampled. This occipital head region does not have skin scales and hence better allows preparation of skin sections suitable to demonstrate subtle damages to the skin histology. The samples (D2; *n* = 12) were collected from randomly selected fish, i.e., three from each group sampled by either the absorption, wiping or scraping method, as well as from three fish unexposed to prior mucus sampling. All skin samples were fixed in buffered formalin (10%) for 24 h, then transferred to ethanol (70%) and stored at 4 °C until embedded in paraffin blocks. Sections of 3 µm were placed on Superfrost Plus^®^ glass slides (ThermoFisher Scientific, Waltham, MA, USA), and left to dry at 37 °C for 12 h. To identify the mucus cells in the skin epidermis, the slides were stained with Alcian blue-periodic Acid Shift and counterstained with hematoxylin. A bright-field optical microscope (Leica DM2000 LED; Leica Microsystems CMS GmbH, Wetzlar, Germany) was used to view the slides (*n* = 36), i.e., three from each skin sample. The invasiveness of the three skin mucus sampling methods was compared by semi-quantitative evaluation of the histological alteration to the integrity of the skin epidermis after the mucus sampling. Specifically, skin squamous epithelial cell lining (SECL) disruption and the number of epidermal mucous cells in the skin epidermis/microscopic field after application of each mucus-sampling were used as a measure of invasiveness. On each slide, four microscopic fields were examined, the average of epidermal mucous cell number per the four fields was calculated, and SECL disruption score was given according to the scoring scale defined in Table 1.

### 2.5. ELISA Analyses and Development of a Sensitive ELISA

A conventional indirect ELISA was used to measure specific IgM-A (in serum samples) against different bacteria included in the ALPHA JECT vaccine, in principle following ELISA protocols described previously [22,23,24], and total C5 was measured in skin mucus samples using a sandwich ELISA (Table 2). To improve the detection sensitivity, an enzyme-cascade-amplification strategy colorimetric immune assay (ECAS-CIA) protocol was developed by modifying the protocol of Gao et al. [20]. Briefly, after washing off unbound secondary Ab (as in a conventional ELISA), plates were incubated with 50 µL of tertiary Ab (Goat Anti-mouse IgG H+L ALP, Abcam ab97020; 1:2000) for 1 h at RT. Unbound tertiary Ab was washed off, and 20 µL of 0.05 M carbonate buffer (pH 9.8) containing 5.0 mM anhydrous ascorbic acid 2-phosphate (AA-P) (Sigma, A4403) and 1.0 mM MgCl_2_ were added per well. Plates were incubated at 37 °C for 20 min before a freshly-made mix of 3 µL of water-soluble gold nanoparticles in citrate buffer (AuNP: 15 nm, STREM chemicals, 79-0186), 3 µL of fresh-made 1.5 mM potassium hexachloropalladate solution (Sigma, 334502), 12 µL of 0.1 M HCl solution and 9 µL of 0.1 M Phthalate buffer (pH 4.0) were added to each well. Plates were incubated at 37 °C for 10 min, and notably not washed, before adding 150 µL chromogenic substrate, made by mixing 1-StepTM Ultra TMB-ELISA (Thermo Fisher Scientific, 34029, Rockford, IL, USA) and 50% H_2_O_2_ (Thermo Fisher Scientific, 10185790) in a ratio of 2:1. Color was developed for 10 min at 37 °C and stopped by adding 50 µL of 2 M H_2_SO_4_, and absorbance read at 450 nm using an ELISA plate reader (Multiscan EX, Artisan; Thermo Electron Corporation, Vantaa, Finland). The details of the ECAS-CIA protocol used are given in Table 2. Negative (a pool of sera from unvaccinated fish), positive (a pool of sera from vaccinated fish), and blank controls (wells without coat, primary Ab and secondary Ab, respectively) were included in each ELISA and ECAS-CIA run. Wells with optical density (OD) values ≥ 3 standard deviations above the negative control values were considered positive wells.

The sensitivity of ECAS-CIA and conventional ELISA was compared by measuring the signal intensity (optical density) produced by the methods. Serum samples (D2; *n* = 28) from vaccinated fish were analyzed for *A. salmonicida*-specific IgM-A OD values by ECAS-CIA and ELISA at 1:1000, 1:2000 and 1:4000 dilutions, respectively. In addition, sera and mucus from four fish were serially diluted (serum: 1:500 to 1:16,000; mucus: 1:2 to 1:128) and analyzed by ELISA and ECAS-CIA to compare the signal intensity at higher dilutions.

### 2.6. Abundance of Immune Molecules in Skin Mucus Collected by Different Methods

The abundance of *A. salmonicida*-specific IgM-A and C5 in skin mucus samples (D2; *n* = 14) obtained using the different mucus-sampling methods was measured using ECAS-CIA and ELISA, respectively.

### 2.7. Abundance of Immune Molecules in Absorbed Mucus from Different Skin Regions

Skin mucus samples from the head, trunk, and tail region of fish (D1; *n* = 19) were collected using the absorption method and compared in terms of their abundance of *A. salmonicida*-specific IgM-A and C5 using ECAS-CIA and ELISA, respectively.

### 2.8. Specific Immune Response to Vaccine in Absorbed Gill Mucus

We used the ECAS-CIA protocol to investigate whether specific IgM-A antibodies against *A. salmonicida* and *V. salmonicida* were detectable in the absorbed gill mucus samples (D2; *n* = 28) collected from the vaccinated fish.

### 2.9. Effect of Absorptive Material on Yield of Immune Molecules in Absorbed Mucus

Different materials used for absorbing skin mucus could differ in their ability to collect and release the individual components of the mucus. To investigate this, a total amount of 1200 µL from each scraped mucus sample (D2; *n* = 20), was subdivided into four aliquots of 300 µL. Three of the aliquots were separately loaded into the upper compartment of a Spin-X tube stuffed with either medical wipes (2.5 × 7 cm), gauze (2.5 × 7 cm; Norgesplaster, Vennesla, Norway), or cotton (0.11 g, which is equivalent to the weight of one piece of medical wipe; AAH pharmaceuticals Ltd., Ruislip, UK). The fourth aliquot was passed through a Spin-X tube without an absorbing material. After collecting mucus as described earlier, we used ECAS-CIA and ELISA, respectively, to measure *A. salmonicida*-specific IgM-A antibodies and C5 in the control aliquot and the three test aliquots from each of the 20 samples.

### 2.10. Statistics

Non-parametric tests were used to perform the statistical analyses when the data were found to violate the assumptions of parametric tests. We used a Wilcoxon signed-rank test and the non-parametric multiple comparisons test (Wilcoxon each pair) to determine the significance of the differences between the means of two and three groups, respectively. A Kendall rank correlation test was used to correlate different ELISA OD values, and coefficient values (τ) were calculated. JMP software (JMP^®^, Version 11, SAS Institute Inc., Cary, NC, USA, 1989-2007) was used to perform all the statistical analyses in this study, with the α value set to 0.05. All graphs were made using GraphPad Prism (GraphPad Software, www.graphpad.com).

## 3. Results

### 3.1. Application of Enzyme-Cascade Amplification Increases ELISA Sensitivity

**Figure 2:**
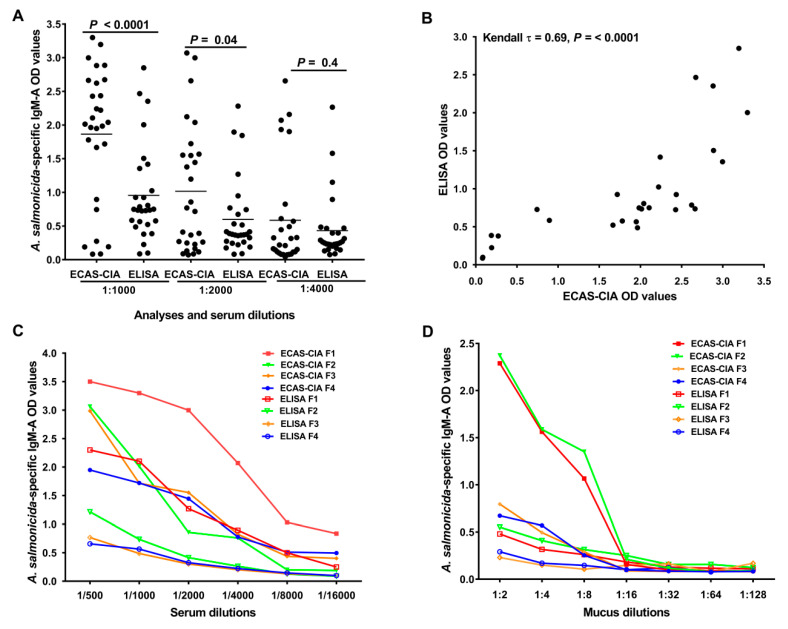
Establishment of the ECAS-CIA method using serum and mucus samples from Atlantic salmon. (**A**) Scatter dot plot comparing signal intensities of ECAS-CIA and ELISA after screening serum samples (D2; *n* = 28) at different dilutions for specific IgM-A response against *A. salmonicida* antigens found in the ALPHA JECT vaccine. Horizontal lines represent the median OD values in different groups. *p*-values were calculated using a Wilcoxon signed-rank test. (**B**) Kendall’s rank correlation test for association between ECAS-CIA and ELISA OD values (450 nm) of *A. salmonicida*-specific IgM-A in serum samples (at a 1:1000 dilution) from vaccinated salmon (D2; *n* = 28). Titration curves comparing the sensitivity of ECAS-CIA and ELISA methods using (**C**) serum and (**D**) mucus samples collected from four fish (F1–F4) with different ELISA OD values. In these titration curves *A. salmonicida*-specific IgM-A OD values (450 nm) were measured in samples with different dilutions.

We assessed the sensitivity of ECAS-CIA and conventional ELISA by comparing the signal intensity (optical density) produced by the two methods in serum and mucus. Analysis of serum for *A. salmonicida*-specific IgM-A showed that ECAS-CIA OD values were significantly higher than those of ELISA at serum dilutions of 1:1000 and 1:2000, but not at 1:4000 (*p* < 0.0001, *p* = 0.04, and *p* = 0.4, respectively; Figure 2A). A Kendall’s rank correlation analysis of the ECAS-CIA and ELISA OD values at 1:1000 dilution showed a significant positive correlation (Kendall τ = 0.69, *p* < 0.0001; Figure 2B) between the produced OD values, suggesting a good agreement between the methods. Analyses of serial dilutions of selected serum (Figure 2C) and absorbed skin mucus (Figure 2D) samples at different dilutions showed gradual decreasing signals for both methods, but across the different dilutions the ECAS-CIA signals were generally higher than those of ELISA. The analyses of mucus and serum samples showed higher specific IgM-A positive signals at a dilution up to 1:8 and 1:4000, respectively. Due to a robust signal level, the 1:2 and 1:1000 dilution of mucus and serum samples, respectively, were used in all the subsequent experiments.

### 3.2. Vaccination Induces Antigen-Specific Antibodies in Scraped Skin Mucus

**Figure 3:**
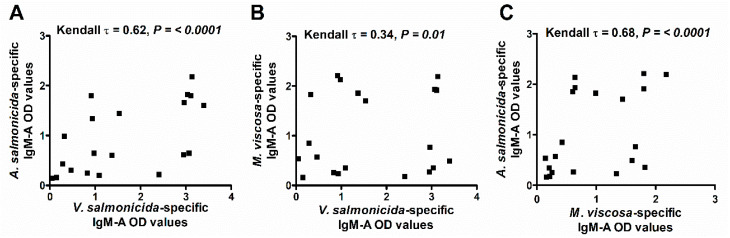
Scatter plots of specific IgM-A antibody responses (ECAS-CIA OD values) in scraped skin mucus to three different bacterial antigens. The three panels also show the results of Kendall’s rank tests of the correlation (τ) between ECAS-CIA OD values from the analysis against the bacterial antigens. (**A**) *A. salmonicida* versus *V. salmonicida* (**B**) *V. salmonicida* versus *M. viscosa*, and (**C**) *A. salmonicida* versus *M. viscosa*.

We analyzed the scraped skin mucus samples (D2; *n* = 20) for specific IgM-A against *A. salmonicida, V. salmonicida*, and *M. viscosa* using the ECAS-CIA method to evaluate the response to the ALPHA JECT vaccine. The results showed that while some fish had detectable levels of specific antibodies against all three bacteria, others had antibodies against only one or two bacteria, or against none at all (Figure 3). A Kendall’s rank correlation analysis showed that *A. salmonicida*-specific IgM-A OD values correlated positively with those for *V. salmonicida* (τ = 0.62, *p* < 0.0001, Figure 3A) and those for *M. viscosa* (τ = 0.68, *p* < 0.0001; Figure 3C). However, the correlation was weak between *V. salmonicida* and *M. viscosa* specific IgM-A OD values (τ = 0.34, *p* < 0.01; Figure 3B). Given that *A. salmonicida* specific Ab correlated with antibodies against the two other bacteria, only the specific antibody response against *A. salmonicida* was analyzed in the remaining experiments involving absorbed skin mucus samples.

### 3.3. Abundance of Immune Molecules in Skin Mucus Collected by Different Methods

**Figure 4:**
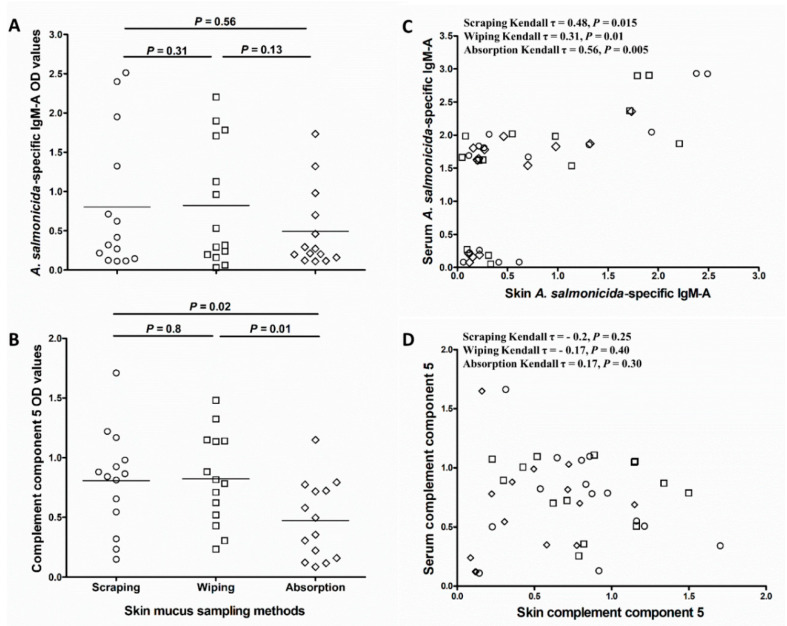
Influence of skin mucus sampling method on the downstream analysis of specific IgM-A and complement component 5 in skin mucus. Scatter dot plots comparing (**A**) *A. salmonicida*-specific IgM-A OD values (obtained using ECAS-CIA) and (**B**) complement component 5 OD values (obtained using ELISA) of mucus samples collected by the three different methods (D2; *n* = 14). The nonparametric multiple comparisons test (Wilcoxon each pair) was performed to calculate the significance of the OD value differences between the three methods. Horizontal lines in “A” and “B” represent the median OD values in different groups. Kendall’s rank test of correlation (τ) between (**C**) *A. salmonicida*-specific IgM-A or (**D**) complement component 5 OD values in skin mucus (collected by the three different methods) and the corresponding values in serum samples.

Comparison of antigen-specific IgM-A and C5 abundance in the different skin mucus sample types showed that OD values for *A. salmonicida*-specific IgM-A and C5 were lower in absorbed samples compared to wiped and scraped samples. Although the differences were not statistically significant for *A. salmonicida*-specific IgM-A (*p* = 0.31 and 0.56, respectively; Figure 4A), they were significant for C5 (*p* = 0.01 and 0.02, respectively; Figure 4B). Kendall’s rank correlation analysis of *A. salmonicida*-specific IgM-A OD values in skin mucus samples collected in a pairwise fashion from the same fish showed relatively strong correlation between wiped and scraped samples (Kendall τ = 0.81, *p* = 0.01; Figure S1A), and between wiped and absorbed samples (Kendall τ = 0.71, *p* = 0.02; Figure S1C). The correlation between absorbed and scraped *A. salmonicida*-specific IgM-A OD values was less strong and only near significant (Kendall τ = 0.61, *p* = 0.05; Figure S1B). In addition, *A. salmonicida*-specific IgM-A OD values in absorbed mucus correlated moderately (Kendall τ = 0.56, *p* = 0.005 Figure 4C) with those of serum samples from the same fish. Though statistically significant, the correlation with serum samples were weaker for both wiped and scraped mucus samples (Kendall τ = 0.31, *p* = 0.01 and Kendall τ = 0.48, *p* = 0.015, respectively; Figure 4C). Kendall’s rank correlation analysis of OD values for C5 showed that there were no correlations between C5 levels in any of the skin mucus sample types and serum (scraping; Kendall τ = −0.2, *p* = 0.25, wiping; Kendall τ = −0.17, *p* = 0.4, and absorption; Kendall τ = 0.17, *p* = 0.3) (Figure 4D).

### 3.4. Mucus Sampling by Absorption Causes Little Damage to the Mucosal Epithelium

**Figure 5:**
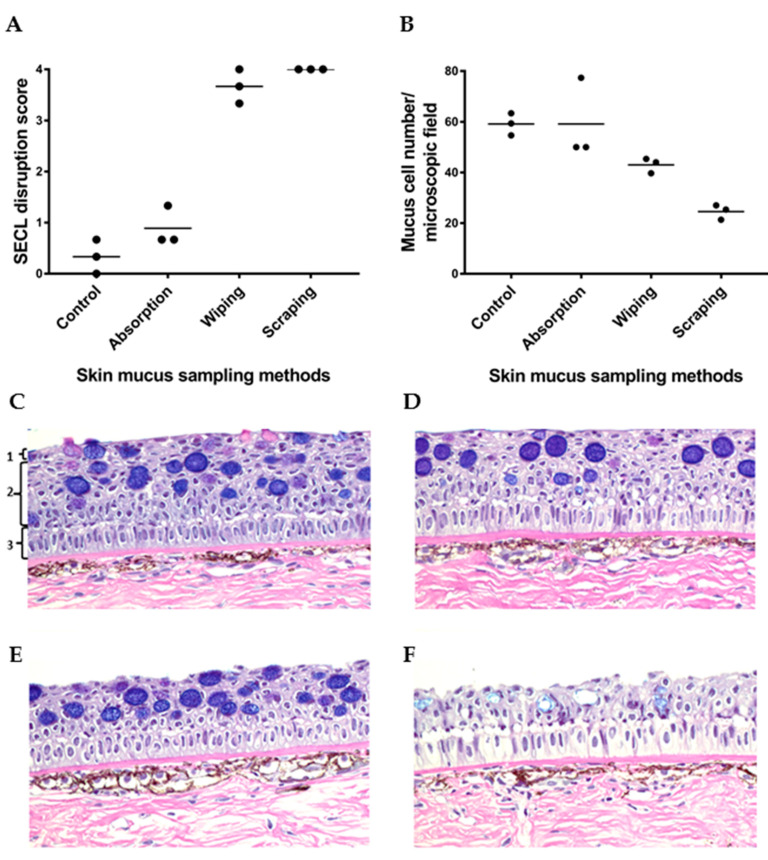
Evaluation of the invasiveness of three skin mucus sampling methods (absorption, wiping and scraping) to salmon skin after mucus sampling. (**A**,**B**) Comparison of SECL disruption score (**A**) and mucous cell number in skin epidermis/microscopic field (**B**) between skin mucus sampling methods after mucus sampling. Horizontal lines represent the median in the different groups of samples. (**C**–**F**) Histology sections of salmon skin samples from occipital head region stained with Alcian blue/periodic acid-Schiff showing the effect of different skin mucus sampling methods on the integrity of the skin epidermis. (**C**) Control fish skin showing the normal structure of salmon skin, as well as the following epidermal layers: (1) squamous epithelial cell lining, (2) cuboidal epithelial cell layers, and (3) basal columnar epithelial cell layer. The remaining histological images show that the sampling methods differ in the extent to which they disrupt the epidermis, with the absorption method (**D**) being the least disruptive to the three epithelial cell layers indicated in panel A, followed by the wiping method (**E**), and finally the scraping method (**F**) being the harshest to them. The microscopic images in (**C**–**E**) and F were taken of skin section with 0, 1, 3 and 4 SECL disruption score, respectively. All images were captured at 40× magnification.

The invasiveness of the mucus sampling methods was evaluated by assessing the histological disruption of squamous epithelial cell lining of skin epidermis, and the number of epidermal mucous cells/microscopic field after mucus sampling (Figure 5). Salmon skin without prior mucus sampling showing standard histological structure of salmon skin was used as a control sample (Figure 5C). The histological analysis of skin samples collected after mucus absorption indicated that the absorption method was the least invasive of all the methods, as evidenced by the three intact skin epidermal layers (Figure 5D). Next was the wiping method (Figure 5E), where only the upper layers of the epidermis were damaged after sampling, including the SECL and part of the cuboidal epithelial cell layers. The most invasive approach was the scraping method, which led to an erosion of most of the epithelial cell layers (Figure 5F). The SECL disruption score of absorbed skin samples was comparable to skin samples without prior mucus sampling and was on average lower than that of wiped and scraped skin (Figure 5A). The observation that the number of epidermal mucous cells (shown as dark blue cells in Figure 5) in the scraped skin samples was the lowest in comparison to the control, absorbed and wiped skin samples (Figure 5B), further corroborated the former as being the most invasive method.

### 3.5. Levels of Immune Molecules in Skin Absorbed Mucus Are Influenced by Region Sampled

**Figure 6:**
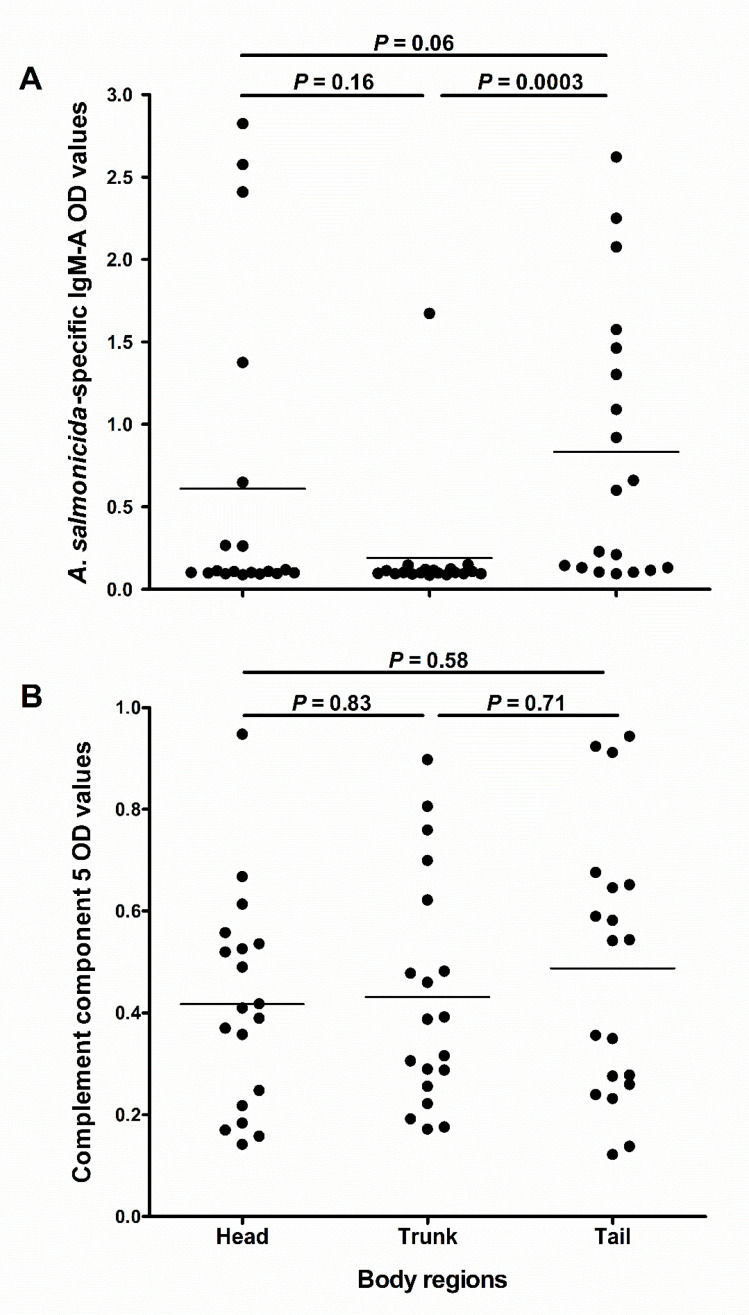
Abundance of immune molecules in mucus from different skin regions of salmon. The scatter dot plots compare (**A**) the *A. salmonicida*-specific IgM-A OD values (measured using ECAS-CIA) and (**B**) complement component 5 OD values (measured using ELISA) in absorbed skin mucus samples (D1; *n* = 19) obtained from the head, trunk and tail regions of fish. Horizontal lines represent the median OD values in the different groups of samples. The nonparametric multiple comparison test (Wilcoxon each pair) was used to calculate the *p* values.

We investigated whether the abundance of C5 and IgM-A would differ in mucus sampled from different skin regions. The results showed that IgM-A OD values in the tail region were near statistically different from those in the head region (*p* = 0.06; Figure 6A), and statistically higher than in the trunk region (*p* = 0.0003; Figure 6A). However, there was no statistical difference between IgM-A OD values in the head and trunk region (*p* = 0.16; Figure 6A). There were no significant differences in C5 OD values between the three sampled regions (Figure 6B).

### 3.6. Specific Immune Response to Vaccine in Absorbed Gill Mucus

The ECAS-CIA analysis of specific antibody response in the absorbed gill mucus to vaccination with ALPHA JECT showed that 28.5 and 39.2% of the samples were positive (i.e., responders) for specific IgM-A against *A. salmonicida* and *V. salmonicida*, respectively. In comparison, 85.7 and 57.0% of the serum samples from the same fish had specific IgM-A against *A. salmonicida* and *V. salmonicida*, respectively (Figure S2A). A Kendall’s rank correlation analysis of *V. salmonicida* or *A. salmonicida*-specific antibodies in gill mucus with the corresponding values in serum samples showed a weak correlation in the case of *V. salmonicida* (Kendall τ = 0.33, *p* = 0.01 Figure S2B), but not in the case of *A. salmonicida* (Kendall τ = 0.23, *p* = 0.08; Figure S2B).

### 3.7. Effect of Absorptive Material on Yield of Immune Molecules in Absorbed Mucus

**Figure 7:**
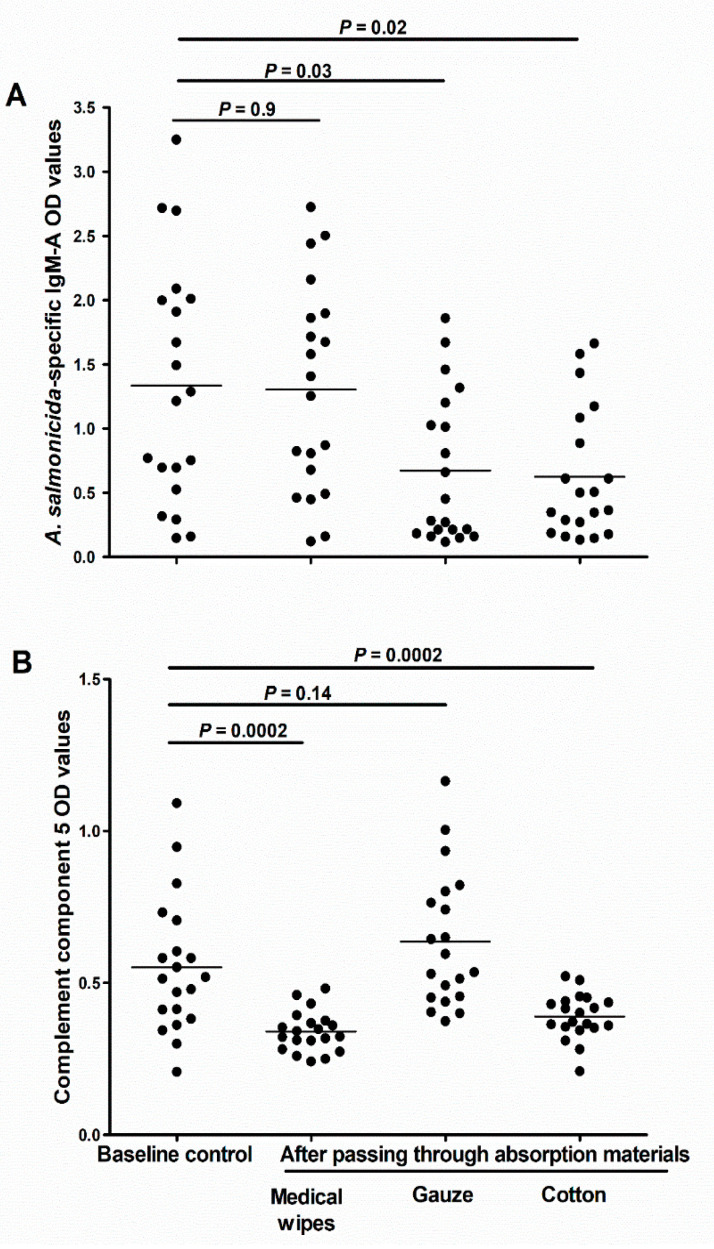
Effect of different mucus absorbing materials on the downstream analyses of the skin mucus samples. (**A**) Scatter dot plot of *A. salmonicida* specific IgM-A OD values (measured using ECAS-CIA), and and (**B**) complement component 5 (measured using ELISA) in scraped skin mucus samples (D2; *n* = 20) before and after passing the samples through either medical wipes, gauze, or cotton. Horizontal lines represent the median OD values in the different groups of samples. The nonparametric multiple comparison tests (Wilcoxon each pair) was used to calculate the *p* values.

We investigated whether different absorptive materials could vary in their ability to collect and release the individual components of the mucus. Our results showed that in comparison to the control samples (spun over no absorptive material), the aliquots passed through cotton or gauze had significantly less specific IgM-A (*p* = 0.03 and 0.02, respectively; Figure 7A). Interestingly, there was no difference in the amounts of IgM-A between the baseline aliquots and aliquots passed through medical wipes (*p* = 0.9; Figure 7A). In contrast, we found significantly less C5 after passing the mucus through medical wipes or cotton (*p* = 0.0002 for both; Figure 7B), but no significant difference after passing it through gauze (*p* = 0.14; Figure 7B).

## 4. Discussion

The study of immune components in fish skin mucus stands to enrich our understanding of the host’s first line of defense against pathogens. Naturally, the first step in initiating such a study is sampling for mucus. In this and two other companion metabolomics [10] and proteomics [11] studies, we evaluated and compared a so-called “absorption” method against two commonly used methods for sampling skin mucus, namely, “wiping” and “scraping”. The comparison made in the current study focused primarily on the use of specific methods that allows quantification of two specific immune proteins in skin mucus samples. The invasive nature of these mucus sampling methods on skin integrity was also compared.

Firstly, we investigated antigen-specific IgM-A, a component of the adaptive immune system that is routinely assayed to gauge the success of vaccination procedures in Atlantic salmon. In addition, we also investigated C5, an important innate humoral immune molecule that plays a pivotal role in the inflammation process [13]. In anticipation of low levels of IgM in mucus, we adapted and developed a sensitive ECAS-CIA method that facilitated the analysis of this immune molecule.

Secondly, we hypothesized that different approaches to collect skin mucus from fish might impact the integrity of fish skin differently. In a study by Raj et al. [25], it was shown that using different materials to rub fish to collect mucus could remove the uppermost layers of epidermal cells. Here, the histological analysis of skin regions after mucus had been sampled by either absorption, wiping or scraping, showed that while the scraping method was the most invasive to skin epidermis, the absorption method was the least invasive. Given that the absorption method least impairs fish skin—and presumably, the well-being of the fish—fish from which skin mucus has been sampled using the absorption method will potentially fare better when kept alive, such that serial sampling could be performed to measure various parameters in skin mucus over time. The absorption method is, therefore, the sampling approach that better meets the refinement and reduction principles of the 3Rs principles of using fish as laboratory animals.

Besides affecting the integrity of fish skin, it is likely that the different mucus sampling methods could generate mucus samples that differ both quantitatively and qualitatively. This notion is supported by metabolomics [10] and proteomics [11] analyses previously performed on mucus samples used in this study. For example, when compared to the scraped and wiped samples, the absorbed mucus was found to be less contaminated with intracellular proteins that most likely would have originated from damaged epidermal cells [11]. Given such nuances, we investigated the impact of the three sampling methods on immune molecules in the mucus samples. Specifically, the abundance of C5 and antigen-specific IgM-A in mucus from vaccinated fish were compared. The results showed that levels of *A. salmonicida*-specific IgM-A in the absorbed mucus samples were comparable to those in the wiped and scraped samples, showing the appropriateness of the absorption method to collect skin mucus samples for antigen-specific IgM assay in fish. All fish used for the antigen-specific IgM assay in the current study were as big as 1–2 kg; however, the possibility of absorbing skin mucus samples from small fish (down to 50 gm) for the same purpose was also confirmed in a pilot study (data not shown).

The levels of *A. salmonicida*-specific IgM-A in mucus samples collected by the three methods showed a significant positive correlation with the corresponding values of the serum samples collected from the same fish. Although IgM antibody in fish skin mucus have been suggested to originate from the blood [6], other data suggest that antibody-producing cells in the skin may contribute [26]. Our observation that some individual fish had antigen-specific IgM-A in serum but not in mucus, similar to that reported by Hatanaka et al. [27], supports the notion that the two compartments may operate with a certain degree of independence. The abundance of C5, on the other hand, was significantly lower in absorbed mucus in comparison to in the wiped and scraped samples. The fact that the passage of mucus through medical wipes did not reduce levels of IgM-A, but did reduce levels of C5, could potentially explain the different outcome for the two proteins. If so, the use of more optimal absorption materials for C5, such as gauze, could improve the efficiency of the absorption method for this protein. The lack of correlation between C5 abundance in skin mucus (collected by any of the three methods) and serum suggests that levels of C5 in blood cannot be directly estimated by analysis of skin mucus. To the best of our knowledge, no studies have previously presented quantitative data regarding potential correlation of C5 protein between serum and mucus in fish. The discrepancy found between C5 levels in skin mucus and serum in the current study could then suggest that the two compartments are supplied with C5 protein from different tissue sources. The C5 population in skin mucus could, for example, be partially supplied by production in dermal macrophages [28].

We also observed that different absorptive materials could vary in their ability to absorb and/or release C5 and specific IgM-A molecules from skin mucus. Although we offer no explanation for these differences—outside of speculating that physicochemical interactions may have been at work—we suggest, however, that pilot studies first be performed to determine the suitability of a particular absorptive material for the collection of the desired immune molecule from mucus samples. In our case, medical wipes were ideal for IgM-A analysis, whereas gauze was ideal for C5 analysis.

So far, our discussion has centered on observations made using mucus samples collected from the lateral sides of salmon. As judged by transcript levels of several immune genes, skin areas from different fish body regions seem to be immunologically diverse [29,30]. In this study, the comparison between different skin regions was performed at the protein level by measuring the abundance of antigen-specific IgM-A and C5 in absorbed mucus samples collected from the head, trunk and tail regions of salmon. Our results showed that antigen-specific IgM-A from the tail region was significantly higher than that collected from the trunk region, whereas the abundance of total C5 was not significantly different between the different regions. The difference in the distribution of C5 and antigen-specific IgM-A between fish skin regions suggests that the immunocompetence of mucus in these regions might also vary. It is important to frame this observation in the context of why some pathogens can breach the mucus defense and induce disease in some areas more so than in others. *Yersinia ruckeri*, for example, causes Red mouth disease [31] in the mouth region of rainbow trout, and *Aeromonas hydrophila* causes tail rot [32] in the tail region of salmonids. In these particular diseases, it would be interesting to employ the absorption method and its ability to collect mucus from pre-selected skin regions, to compare skin mucus composition between the healthy and diseased skin regions.

A preliminary investigation of the potential of using the minimally invasive nature of the absorption method to sample mucus from gill tissue was made. Compared to serum, fewer absorbed gill mucus samples were positive for specific antibodies to *A. salmonicida* and *V. salmonicida* (28.5 and 39.2% versus 85.7 and 57.0%), and there were only weak correlations between these values in serum and gill mucus. Whether this discrepancy reflects a biological difference or is caused by incomplete sampling of gill mucus remains uncertain. The invasiveness of the absorption protocol when applied to gill epithelium was not investigated by histology, but visual inspection of applied wipes suggested that the method, following careful optimization, can be useful for future sampling from this delicate tissue.

The absorption method is an effortless and time-saving approach that could be used to collect fish skin mucus in a minimally invasive manner, with the additional benefit of allowing the possibility of safe multiple sampling from the same fish, from very specific sampling sites. The ease with which this method could be applied on gills and skin encourages future studies to compare the mucus content in these two mucosal surfaces.

## 5. Conclusions

This study is the second of three companion papers that compare three skin mucus sampling methods (absorption, wiping and scraping). Our data substantiate the absorption approach as a minimally invasive sampling method that best maintains the integrity of skin epidermis largely unchanged. The high sensitivity of the ECAS-CIA method facilitated the analysis of low abundant molecules, such as antigen-specific IgM-A, in absorbed mucus samples better than standard ELISA. Among the samples obtained using the mucus-sampling methods tested, absorbed skin mucus samples were found to contain comparable amounts of antigen-specific IgM-A but significantly less C5 when compared to wiped and scraped mucus samples. However, different mucus absorbing materials varied in their ability to absorb and/or release different components in mucus. Lastly, the difference in abundance between IgM-A and C5 in absorbed mucus from different skin areas suggests that careful consideration should be taken when selecting a skin body region for mucus sampling.

## Figures and Tables

**Table 1:** Scoring scale of squamous epithelial cell lining (SECL) disruption in salmon skin after skin mucus sampling.

SECL Disruption Score	Score Description
0	Intact SECL in four out of four microscopic fields
1	Intact SECL in three out of four microscopic fields
2	SECL is intact in two fields and disrupted in two fields
3	Disrupted SECL in three out of four microscopic fields
4	Disrupted SECL in four out of four microscopic fields

**Table 2:** Specifications of Enzyme-Linked Immunosorbent Assay (ELISA) and enzyme-cascade-amplification strategy colorimetric immune assay (ECAS-CIA) analyses.

	Enzyme-Linked Immunosorbent Assay (ELISA)		Enzyme-Cascade-Amplification Strategy Colorimetric Immune Assay (ECAS-CIA)
Immune assay	C5 Sandwich ELISA	Specific IgM-A	Specific IgM-A
Coating	Mouse mAb against Atlantic Salmon C5; (0L-3A11) (50 µL of 0.5 µg/mL)	Sonicated *A. salmonicida* (50 µL of 5 µg/mL)	Sonicated bacteria, 50 µL of *A. salmonicida* (5 µg/mL), *V. salmonicida* (1 µg/mL) and *M. viscosa* (5 µg/mL)
Washing buffer	PBS	PBS with 0.1% Tween-20 (200 µL) ^1^	
Blocking buffer	PBS.BSA (2.5%)	PBS with 5% BSA (200 µL) ^1^	
Detection Ab	Mouse mAb against Atlantic Salmon C5 (0L-20C12) (0.5 µg/mL).	Mouse mAb against Salmonid-IgM-A (4C10, 1:20 in 0.25% BSA.PBS) (50 µL) ^1^	
Secondary Ab conjugate	Goat anti-mouse IgG1 HRP, Antibodies-online, BIN577017; 1:4000; in 0.25% BSA.PBS	ECL^TM^ sheep Anti-mouse IgG HRP, GE Healthcare, NA931V; 1:2000 in 0.25% BSA.PBS (50 µL)	Goat Anti-mouse IgG H+L ALP, Abcam ab97020; 1:2000 (50 µL)

^1^ These treatments were applied in specific immunoglobulin M subtype A (IgM-A) analyses with ELISA and ECAS-CIA.

## Supplementary Materials

The following is available online at https://www.mdpi.com/2076-2615/10/8/1374/s1, Figure S1: Scatter plots, including Kendall’s rank correlation analyses of *A. salmonicida*-specific IgM-A OD values between skin mucus samples collected by absorption, wiping and scraping, in a pairwise fashion from the same fish. **Figure S2**: Specific immune responses against ALPHA JECT vaccination in fish absorbed gill mucus and serum.
