# Supplementary material for: Quantification of Defensive Proteins in Skin Mucus of Atlantic salmon Using Minimally Invasive Sampling and High-Sensitivity ELISA
Source: Animals (Basel). 2020 Aug 7;10(8):1374. doi: 10.3390/ani10081374 (PMC7460368; doi:10.3390/ani10081374)
Supplement: Supplementary file 1 [file animals-10-01374-s001.pdf]

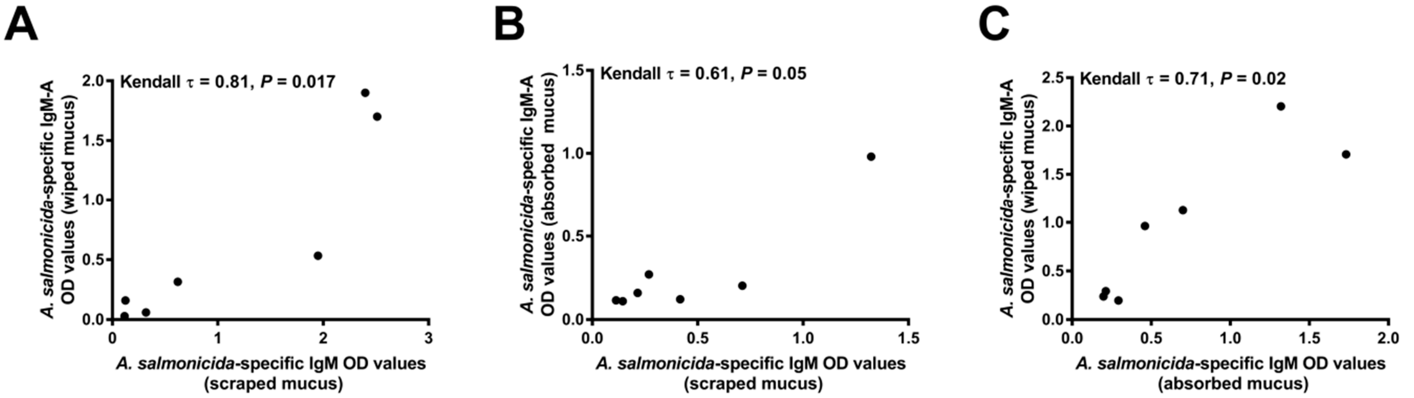

Supplementary Figure 1. Scatter plots, including Kendall's rank correlation analyses of *A. salmonicida*-specific IgM-A OD values between skin mucus samples collected by absorption, wiping and scraping, in a pairwise fashion from the same fish: (A) wiped versus scraped, (B) absorbed versus scraped, and (C) wiped versus absorbed. The Kendall τ and P values are shown.

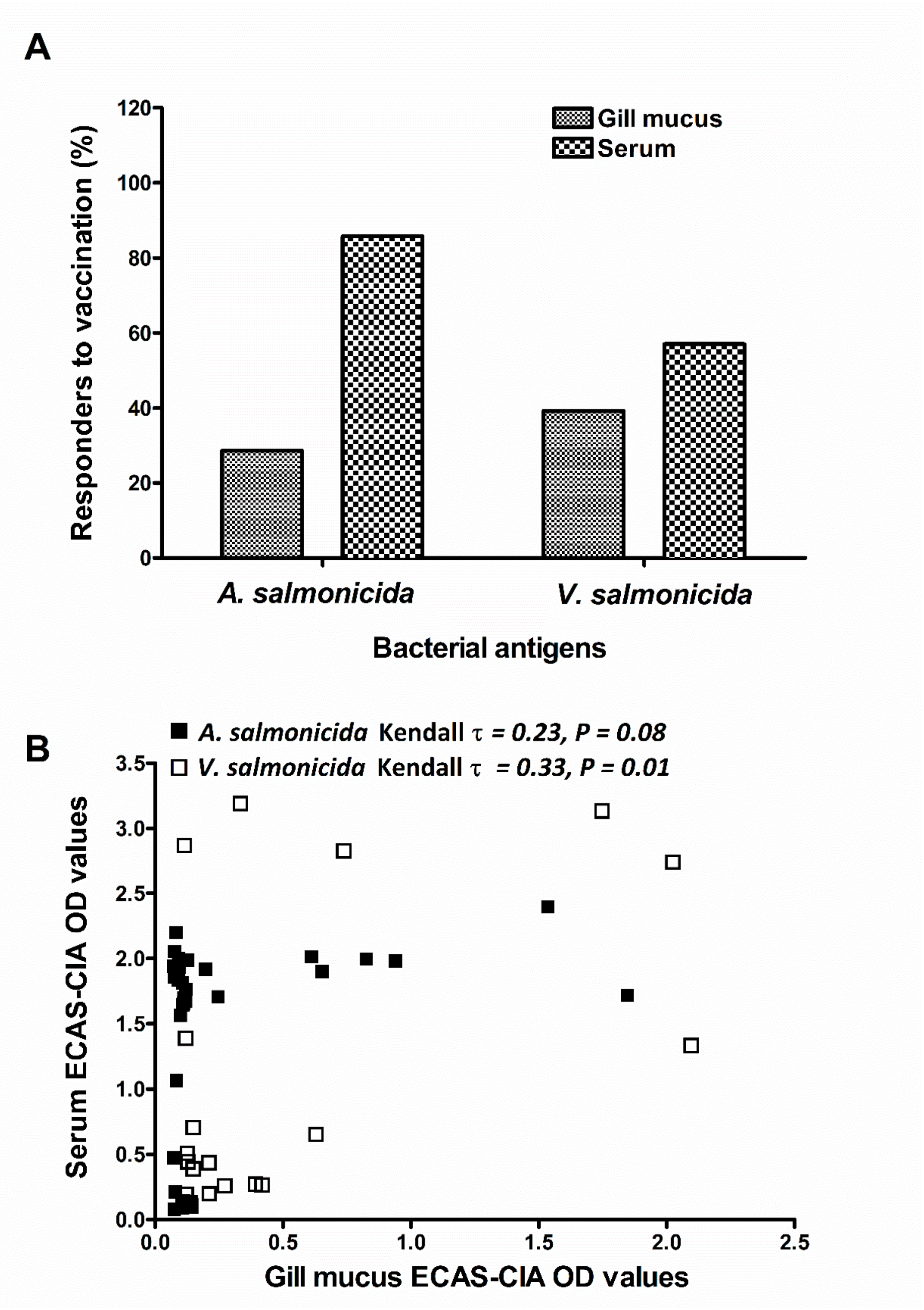

Supplementary Figure 2. Specific immune responses against ALPHA JECT vaccination in absorbed gill mucus and serum. from Atlantic salmon. (A) Shows the percentage of responder fish to vaccination based on ECAS-CIA positive signals for antigen-specific IgM-A in absorbed gill mucus and serum (D2; n = 28) against both *A. salmonicida*- and *V. salmonicida*. (B) Shows Kendall's rank correlation analyses between the *A. salmonicida*- or *V. salmonicida*-specific IgM-A OD values (450 nm) in absorbed gill mucus samples, and the corresponding values in serum samples (D2; n = 28). The Kendall τ and P values are shown.
